# Thermal Stress Disrupts Gut Microbiota, Induces Oxidative DNA Damage, and Modulates Immune and Stress-Related Gene Expression in the Red Sea Urchin (*Loxechinus albus*)

**DOI:** 10.3390/biology15120913

**Published:** 2026-06-11

**Authors:** Katalina Llanos-Azócar, Juan Manuel Estrada, Pablo A. Oyarzún, Phillip Dettleff, Giorgia Daniela Ugarte, Juan A. Valdés

**Affiliations:** 1 Programa de Doctorado en Biotecnología, Facultad de Ciencias de la Vida, Universidad Andres Bello, Santiago 8370186, Chile; k.llanosazcar@uandresbello.edu; 2 Laboratorio de Biotecnología Molecular, Facultad de Ciencias de la Vida, Universidad Andres Bello, Santiago 8370146, Chile; giorgia.ugarte@unab.cl; 3 Interdisciplinary Center for Aquaculture Research—Applied Research (INCAR^2^), Universidad Andres Bello, Santiago 8370146, Chile; mestrada@unab.cl; 4 Centro de Investigación Marina Quintay (CIMARQ), Universidad Andres Bello, Quintay 2340000, Chile; pablo.oyarzun@unab.cl; 5 Escuela de Medicina Veterinaria, Facultad de Agronomía y Sistemas Naturales, Facultad de Ciencias Biológicas y Facultad de Medicina, Pontificia Universidad Católica de Chile, Santiago 7820436, Chile; phillip.dettleff@uc.cl

**Keywords:** *Loxechinus albus*, thermal stress, gut microbiota, oxidative DNA damage, immune and stress response

## Abstract

The red sea urchin *Loxechinus albus* is a species of high commercial importance in Chilean aquaculture, whose performance is strongly influenced by environmental conditions such as temperature. The gut microbiota plays a central role in host physiology; however, its interaction with stress-induced molecular responses remains poorly understood. This study evaluated the effects of thermal stress on food consumption, gut microbial composition, oxidative status, and immune- and stress-related gene expressions in *L. albus* gut. Sea urchins were exposed to control (16 °C) and elevated temperature (22 °C) conditions for 7 and 14 days. Gut microbiota was characterized using 16S rRNA sequencing, while oxidative damage to DNA and proteins was quantified. Gene expression analyses targeted markers of apoptosis (*casp3*, *casp10*, *bak1*), cellular growth (*mtor*, *raptor*), stress response (*hsp70*), and immune regulation (*nfκb*, *foxo*). Thermal stress induced a marked reduction in microbial alpha diversity and promoted a shift toward opportunistic taxa. Heat-stressed individuals exhibited significantly increased oxidative DNA damage, whereas protein oxidation remained unchanged. Gene expression analyses revealed early upregulation of *casp3*, *casp10*, *nfκb*, *foxo*, and *hsp70*, suggesting activation of apoptotic, immune, and stress-response pathways. In contrast, *bak1*, *mtor*, and *raptor* showed limited or no significant modulation. These findings demonstrate that thermal stress disrupts host–microbiota homeostasis and induces oxidative and molecular responses in *L. albus*. This integrative response provides insight into mechanisms underlying physiological performance under thermal stress, with important implications for aquaculture sustainability.

## Simple Summary

Rising seawater temperatures associated with climate change pose a significant challenge for marine aquaculture species such as the red sea urchin *Loxechinus albus*. In this study, we investigated how thermal stress affects food consumption, gut microbiota, oxidative damage, and molecular stress responses. Sea urchins exposed to elevated temperature showed reduced microbial diversity and an increase in opportunistic bacteria, indicating an imbalance in gut microbial communities. In addition, thermal stress caused increased DNA damage and activated genes related to stress response, immunity, and cell death. These findings demonstrate that warming conditions disrupt key physiological processes at multiple levels, from microbiota composition to cellular responses. Understanding these mechanisms is essential for improving the resilience and sustainability of sea urchin aquaculture under climate change scenarios.

## 1. Introduction

Rising seawater temperatures associated with climate change represent one of the most significant stressors affecting marine organisms, particularly in aquaculture systems where environmental conditions can fluctuate and are difficult to control [1]. Temperature is a key abiotic factor shaping physiological performance and microbial dynamics in marine species [2,3]. In recent decades, the increasing frequency, duration, and intensity of marine heatwaves have led to recurrent thermal anomalies in coastal ecosystems worldwide [4]. In sea urchins, elevated temperatures have been associated with alterations in feeding activity, reproductive performance, larval development, immune function, microbiota composition, and species distribution, ultimately affecting population dynamics and ecosystem functioning [5,6]. Thermal stress can disrupt multiple biological processes, ultimately compromising organism health, growth, and survival. Among the systems affected by environmental stressors, the intestinal microbiota plays a fundamental role in host physiology. In marine organisms, gut microbial communities contribute to disease resistance, immune modulation, nutrient absorption, metabolic regulation, vitamin synthesis, and growth [7,8,9,10]. These functions are essential for acclimatization and adaptation under changing environmental conditions [2,11]. However, environmental disturbances such as elevated temperature can alter microbial community structure and function, leading to dysbiosis and reduced host performance [12,13].

In echinoderms, temperature fluctuations have been shown to significantly affect gut microbiota composition, diversity, and stability [14,15]. For example, chronic exposure to elevated temperature modifies the functional potential of the gut microbiome in the sea urchin *Lytechinus variegatus* [15]. Similar patterns have been reported in other marine invertebrates, where thermal stress promotes microbial imbalance and the proliferation of opportunistic taxa, contributing to disease outbreaks and mortality in aquaculture species such as *Crassostrea gigas*, *Panulirus ornatus*, and *Mytilus coruscus* [16,17,18,19]. These findings highlight that disruptions in microbial diversity and community structure can increase host susceptibility to disease [20]. In addition to microbial dysbiosis, thermal stress induces oxidative stress through the excessive production of reactive oxygen species (ROS), which can overwhelm antioxidant defense systems [21]. This imbalance leads to damage in essential biomolecules, including lipids, proteins, and nucleic acids. Among these, oxidative DNA damage, particularly the formation of apurinic/apyrimidinic (AP) sites, is considered a sensitive indicator of cellular stress and genomic instability, while protein carbonylation reflects irreversible oxidative modifications that impair protein function [22]. Despite extensive research on oxidative stress in marine invertebrates, its relationship with microbiota alterations under thermal stress remains poorly understood, especially in echinoderms.

At the molecular level, thermal stress activates conserved pathways involved in stress tolerance and cellular homeostasis. Apoptosis, mediated by caspases, contributes to the removal of damaged cells [23], while the mammalian Target of Rapamycin (mTOR) pathway integrates environmental cues to regulate growth and metabolism [24]. Transcription factors such as Nuclear Factor Kappa-light-chain-enhancer of activated B cells (NFκB) and Forkhead box class O (FOXO) play central roles in immune regulation and oxidative stress responses, linking redox balance with inflammation and antioxidant defenses [25]. The involvement of these pathways in environmental stress responses has been demonstrated in marine invertebrates. For instance, in the blood clam *Tegillarca granosa*, exposure to acidified conditions resulted in significant modulation of genes associated with the NFκB signaling pathway compared to basal conditions [26]. Similarly, early upregulation of *foxo* expression has been reported under stress conditions, which is consistent with its role in activating antioxidant defenses in response to increased oxidative damage [27]. Additionally, heat shock proteins, especially Heat Shock Protein 70 (HSP70), function as molecular chaperones that protect cells from stress-induced protein damage and are widely used as biomarkers of thermal stress in marine organisms [28,29]. In echinoderms, rapid induction of HSP70 has been described as an early response to environmental stress, reflecting its key role in maintaining cellular homeostasis [29,30].

The red sea urchin, *Loxechinus albus* (Molina, 1782), is a species of high commercial importance in Chilean aquaculture, with strong demand in both national and international markets [31,32]. *L. albus* supports one of the most important benthic fisheries in Chile, with average annual landings of approximately 34,000 tonnes between 1981 and 2020 [33]. The species represents a valuable export resource, particularly for Asian markets, and has historically sustained intensive harvesting along the Chilean coast [33]. It also represents a valuable opportunity for diversifying aquaculture production in Chile, which is currently dominated by finfish species [34,35]. The development of sustainable culture practices for *L. albus* could reduce pressure on wild populations while supporting local economies. However, successful cultivation depends on maintaining environmental conditions that ensure optimal physiological performance, where both microbiota stability and stress tolerance mechanisms play critical roles. Despite its ecological and economic relevance, studies addressing the effects of thermal stress on the gut microbiota and associated physiological responses in *L. albus* remain limited. Moreover, most research on microbiome responses to environmental stressors has focused on reef-associated species, leaving temperate species underexplored [15].

In this context, the aim of the present study was to evaluate the effects of elevated seawater temperature on the gut microbiome of *L. albus* under aquaculture conditions. Additionally, oxidative damage biomarkers and the expression of genes associated with stress response (*hsp70*), apoptosis: Caspase-3 (*casp3*), Caspase-10 (*casp10*), BCL2 Antagonist/Killer 1 (*bak1*), and cellular signaling (*nfκb*, *foxo*, *mtor*, *raptor*) were assessed. This integrative approach provides new insights into how thermal stress affects host–microbiota interactions and cellular processes, contributing to a better understanding of the mechanisms underlying resilience and sustainability in sea urchin aquaculture under climate change scenarios.

## 2. Materials and Methods

### 2.1. Red Sea Urchin Maintenance and Experimental Design

The study adhered to animal welfare procedures and was approved by the bioethical committees of the Universidad Andres Bello and the National Agency for Research and Development (ANID) of the Chilean government (protocol code 012/2024, approval date 18 May 2024). One hundred red sea urchins with an average weight of 24 ± 5 g and a length of 4 ± 2 cm were collected from the Quintay Marine Research Center (CIMARQ) and transported to the Laboratory of Reproductive Biology of Marine Invertebrates (33°13′ S, 71°38′ W, Valparaíso, Chile). Sea urchins were divided into two groups: control at 16 ± 2 °C and heat stress at 22 ± 2 °C. The temperature of 22 °C was selected because it represents an extreme thermal condition that may occur during summer thermal anomalies or marine heatwave events in the Valparaíso Region along the Chilean coast [36]. Continuous monitoring confirmed that the experimental groups remained within distinct thermal conditions throughout the study, and no overlap between treatments occurred. Sea urchins were placed in separate 30 L tanks; each group was replicated twice, and each replicate contained 10 experimental individuals. The tanks contained filtered seawater (pH 8 ± 0.2 and salinity 30 ± 1 ppt) under natural photoperiod conditions and constant aeration. Water temperature was maintained using 100 W submersible thermostatic heaters and monitored every 3 h throughout the experimental period using digital thermometers to ensure stable thermal conditions. Nitrate and ammonium concentrations were monitored using commercial colorimetric test kits (HI3874 and HI784, Hanna Instruments, Woonsocket, RI, USA) according to the manufacturer’s instructions. A filter was placed to keep the aquaria clean, and a 30% water exchange was performed to maintain salinity, dissolved oxygen, and pH values constant and nitrate (≤5 ppm) and ammonium (≤0.25 ppm) levels low (Table S1). During the experiment, food consumption was evaluated to assess the impact of elevated temperature on sea urchins. Each tank was divided into two sections, and pre-weighed pieces of *Lessonia spicata* algae, the main component of the natural diet of these juvenile sea urchins, were fed. After 24 h of each trial, total consumption rates were calculated as previously described [37]. At 7 and 14 days into the experiments, 5 individuals from each group were sacrificed, as previously described [10,38]. Briefly, an incision was made in the peristomial membrane to remove Aristotle’s lantern and access the digestive tract. The intestines were rinsed with sterile seawater to remove the intestinal digesta and immediately placed in Eppendorf tubes with RNAlater and stored at −80 °C. Five individuals were collected at each sampling point; however, one sample per group was excluded from downstream analyses due to insufficient nucleic acid quality control criteria. Consequently, four biological replicates were included in the microbiome, oxidative damage, and gene expression analyses.

### 2.2. DNA Extraction, Amplification and Sequencing of 16S rRNA

Metagenomic DNA from sea urchin gut samples was extracted using the ZymoBIOMICS™ DNA Miniprep Kit (Zymo Research, Irvine, CA, USA) (catalog no. D4300) following the manufacturer’s instructions. A library of amplicons of the hypervariable region 4 (V4) of the 16S rRNA gene was created by PCR using specific primers (515F: 5′-GTGCCAGCGCMGCCGCGGTAA-3′ and 806R: 5′-GGACTACHVGGGGGTWTCTAAT-3′) as described in the Earth Microbiome Project [39] using the following amplification conditions: 1 cycle of 94 °C for 3 min, and 35 cycles of 94 °C for 45 s and 50 °C for 60 s and 72 °C for 90 s, and a final extension of 72 °C for 10 min. Libraries were sequenced (2 × 150 bp) utilizing the MiSeq technology (Illumina, San Diego, CA, USA) at the Center for Plant Biotechnology (Universidad Andrés Bello, Santiago, Chile). The raw reads were deposited in the NCBI Sequence Read Archive database (BioProject PRJNA1462746; Biosamples SAMN58996283, SAMN58996284, SAMN58996285, SAMN58996286, SAMN58996287, SAMN58996288, SAMN58996289, SAMN58996290, SAMN58996291, SAMN58996292, SAMN58996293, SAMN58996294, SAMN58996295, SAMN58996296, SAMN58996297, SAMN58996298).

### 2.3. Microbial Diversity Analysis

Raw paired-end 16S rRNA gene sequences were processed using QIIME 2 [40]. Demultiplexed FASTQ files were imported into the QIIME 2 environment. Raw sequencing reads were trimmed by removing low-quality reads (Q < 30) and sequences with lengths less than 30 bp. Quality filtering, paired-end read merging, and chimera removal were performed using the DADA2 plugin. These sequences were subsequently clustered into Operational Taxonomic Units (OTUs) at 97% similarity using the qiime vsearch cluster-features-de novo method to facilitate comparison with traditional microbiome studies. Although DADA2 generates high-resolution amplicon sequence variants (ASVs), OTU clustering at 97% similarity was additionally performed to facilitate comparisons with previous microbiome studies conducted in marine invertebrates and echinoderms. Singleton and low-frequency features were filtered out to reduce potential sequencing artifacts. Taxonomic assignment was conducted using a naïve Bayes classifier trained on the Greengenes2 reference database [41]. Classification was performed using the qiime feature-classifier classify-sklearn method with default parameters. Alpha diversity metrics, including Shannon and Simpson diversity indexes, were calculated using the qiime diversity core-metrics-phylogenetic pipeline. Statistical comparisons of alpha diversity between experimental groups were performed using the non-parametric Kruskal–Wallis test. Taxonomic composition was visualized using stacked bar plots generated with GraphPad Prism v.5.00 (GraphPad Software, San Diego, CA, USA).

### 2.4. Oxidative Damage Assessment

To assess oxidative stress in DNA, apurinic/apyrimidinic (AP) sites were quantified in genomic DNA extracted from 20 mg of gut tissue using the Wizard^®^ Genomic DNA Purification Kit (Promega Corporation, Madison, WI, USA) following the manufacturer’s instructions. DNA concentration and purity were measured with the Epoch Spectrophotometer System (BioTek, Winooski, VT, USA). AP sites were then quantified using the OxiSelect Oxidative DNA Damage Quantification Kit (Cell Biolabs, San Diego, CA, USA), according to the manufacturer’s protocol [42]. Protein carbonylation assay was conducted using total protein extracted from 50 mg of gut tissue homogenized in 1 mL of lysis buffer (50 mM Tris-HCl, pH 7.4; 150 mM NaCl; 1 mM EDTA; 1% NP-40). Samples were solubilized at 4 °C following centrifugation at 12,000× *g*, and protein concentration was determined using the Pierce BCA Protein Assay Kit (Thermo Scientific, Rockford, IL, USA). Protein carbonylation was then quantified using the OxiSelect Protein Carbonyl Spectrophotometric Assay (Cell Biolabs, San Diego, CA, USA), according to the manufacturer’s instructions [42].

### 2.5. Gene Selection, Primer Design and Real-Time qPCR

Candidate genes analyzed under thermal stress were selected through a literature review focusing on key cellular pathways involved in stress responses, including apoptosis, immune regulation, and metabolic signaling. Genes related to heat shock response (*hsp70*), apoptosis (*casp3, casp10, bak1*), and cellular signaling (*nfκb, foxo, mtor, raptor*) were chosen based on their established roles in stress tolerance and physiological regulation in marine invertebrates. Target sequences were obtained from the *L. albus* reference transcriptome, assembled from multiple tissues such as coelomocytes, gonads, and intestine [34]. These sequences were used to design gene-specific primers for mRNA amplification using Primer-BLAST (NCBI, Bethesda, MD, USA, 2.17.0., https://blast.ncbi.nlm.nih.gov/Blast.cgi, accessed on 7 June 2026). Primer quality was further assessed using Oligo Analyzer (Integrated DNA Technologies, Coralville, IA, USA), considering standard conditions (1.5 mM Mg^2+^ and 0.8 mM dNTPs). Potential secondary structures, including hairpins and primer dimers, were evaluated to ensure specificity and amplification efficiency. Primer sequences are provided in Table S2. Total RNA was extracted from gut samples using the TRIzol reagent (Ambion, Carlsbad, CA, USA) following the manufacturer’s protocol. Subsequently, RNA was purified using the RNA Clean and Concentrator™-5 kit (with DNase I) (Zymo Research, Orange, CA, USA), following the manufacturer’s instructions. RNA concentration was determined using a NanoDrop spectrophotometer (Thermo Fisher Scientific, Wilmington, DE, USA), and RNA integrity was assessed by electrophoresis on a 1.2% formaldehyde-agarose gel. For cDNA synthesis, 1 μg of total RNA was reverse transcribed using the ImProm-II Reverse Transcription System (Promega, Madison, WI, USA) according to the supplier’s instructions. Quantitative PCR (qPCR) was performed on a Stratagene MX3000P system (Stratagene, Santa Clara, CA, USA) following the MIQE guidelines [43] using a reaction mix containing 7.5 μL of 2× Brilliant^®^ II SYBR^®^ Green Master Mix (Agilent Technologies, Santa Clara, CA, USA), 6 μL of 40-fold diluted cDNA, and 250 nM of each primer, in a final volume of 20 μL. Negative controls included a no-template control and a no-reverse transcriptase control to check for contamination or genomic DNA amplification. Amplification reactions were performed in triplicate using gene-specific annealing temperatures ranging from 54 to 60 °C, depending on the primer pair. Thermal cycling conditions consisted of an initial denaturation at 95 °C for 2 min, followed by 40 cycles of 30 s at 95 °C (denaturation), 30 s at the corresponding annealing temperature (54–60 °C), and 30 s at 72 °C (extension). The specificity of amplification products was confirmed by a melting curve analysis at the end of each run. The QGene application was utilized to analyze gene expression [44], and data were normalized with 18S rRNA as a housekeeping gene [34].

### 2.6. Statistical Analysis

Prior to analysis, data were assessed for normality and homogeneity of variances using the Kolmogorov–Smirnov and Levene tests, respectively. For oxidative damage biomarkers, differences between control and heat-stressed groups at each sampling point were evaluated using Welch’s *t*-test due to unequal variances, and temporal comparisons within treatments were also performed. Gene expression data obtained by RT-qPCR were analyzed using the non-parametric Kruskal–Wallis test followed by Dunn’s multiple comparisons post hoc test, due to deviations from normality. Statistical significance was established at *p* < 0.05 for all analyses. All statistical analyses were conducted using GraphPad Prism v.5.00 (GraphPad Software, San Diego, CA, USA).

## 3. Results

### 3.1. Food Consumption and Survival

**Figure 1:**
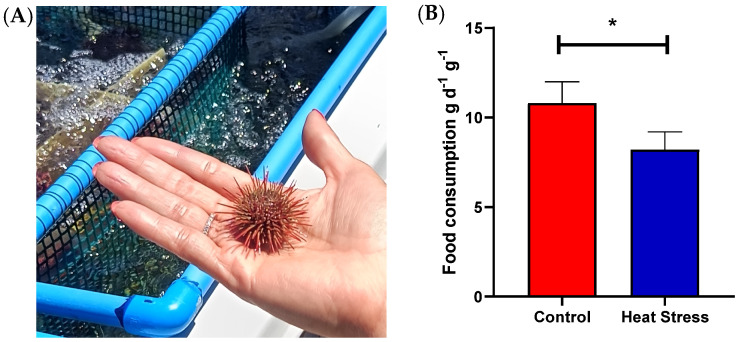
(**A**) Juvenile red sea urchin *L. albus* used in the study. (**B**) Average of food consumption of control and thermal-stressed *L. albus* fed with *Lessonia spicata* algae. Results are expressed as means ± standard error (*n* = 5). Asterisks indicate significant differences between the groups (* = *p* < 0.05).

To evaluate the impact of thermal stress on feeding performance, food consumption was measured in *L. albus* individuals (Figure 1A) maintained under control (16 °C) and elevated temperature (22 °C) conditions during the experimental period. Results showed that sea urchins exposed to control conditions exhibited significantly higher consumption rates compared to those subjected to heat stress (Figure 1B), indicating a negative effect of elevated temperature on feeding activity. No mortalities were observed during the trial. These results indicate that elevated temperature reduced feeding activity in *L. albus* without affecting survival during the experimental period.

### 3.2. Sequencing Data and Diversity of Gut Microbiota

**Figure 2:**
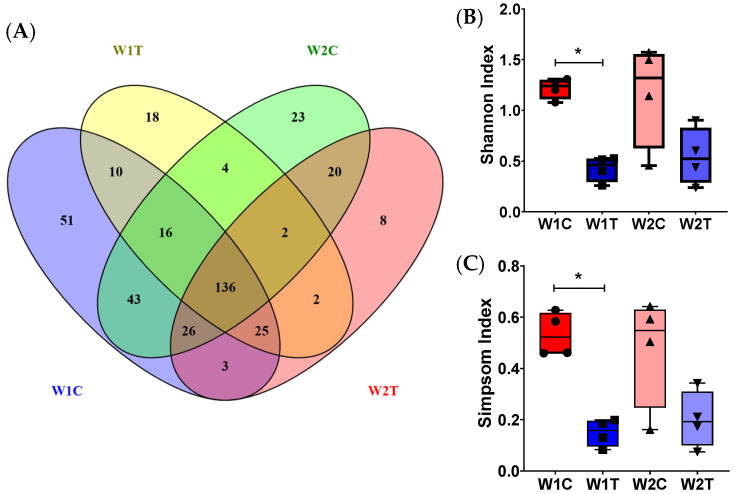
(**A**) Venn diagram depicting the numbers of shared and unique Operational Taxonomic Units (OTUs) among groups W1C (control temperature 16 ± 2 °C, week 1), W2C (control temperature 16 ± 2 °C, week 2), W1T (high-temperature 22 ± 2 °C, week 1), and W2T (high-temperature 22 ± 2 °C, week 2). (**B**) Shannon diversity index among *L. albus* groups. (**C**) Simpson′s diversity index among *L. albus* groups. Shannon and Simpson indexes are expressed as means ± standard error (*n* = 4). Asterisks indicate significant differences between the groups (* = *p* < 0.05).

The microbial communities of *L. albus* gut were determined using 16S rRNA (V4 region). In total, 2,075,150 raw sequences were obtained from 16 samples, and 1,870,964 surviving reads were obtained after quality processing (Table S3). In total, of those reads, 412 OTUs (Operational Taxonomic Units) were identified using a 97% similarity level to analyze the samples. Thermal stress in *L. albus* had a diverse impact on the number of OTUs in their intestines. The four groups shared 136 OTUs in common, with the W1C group showing the highest abundance of unique OTUs (51 OTUs), followed by the W2C group (23 OTUs), the W1T group (18 OTUs), and the W2T group (8 OTUs) (Figure 2A). Significant differences were observed in the alpha diversity indices, including the Shannon index (Figure 2B) and Simpson index (Figure 2C), during week 1, indicating an early effect of the temperature on the structure of the intestinal microbiota. These findings suggest that the initial exposure to experimental conditions induced measurable changes in microbial diversity, reflecting a rapid response of the bacterial community to environmental stress.

### 3.3. Microbial Composition

**Figure 3:**
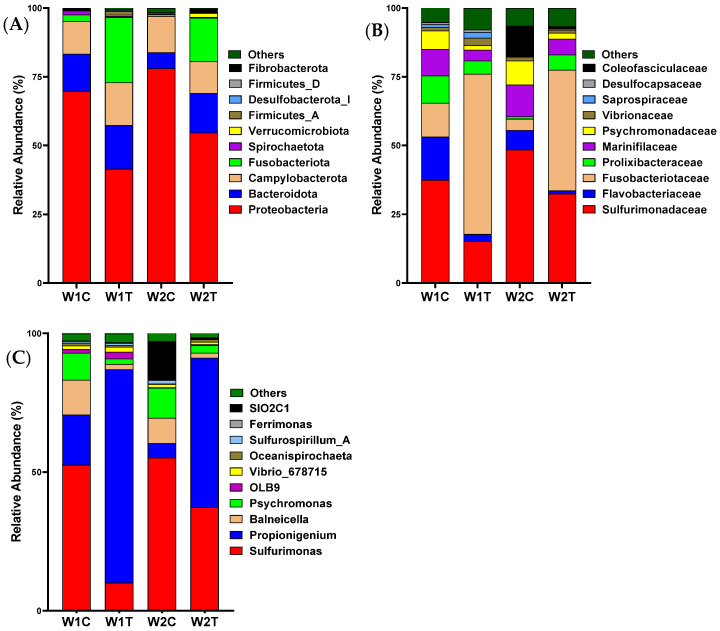
Taxonomic composition of the intestinal microbiota of *L. albus*. (**A**) Relative abundance at the Phylum level, (**B**) Family level and (**C**) at the Genus level. W1C (control temperature 16 ± 2 °C, week 1), W2C (control temperature 16 ± 2 °C, week 2), W1T (high-temperature 22 ± 2 °C, week 1), and W2T (high-temperature 22 ± 2 °C, week 2).

In total, 24 phyla, 44 classes, 81 orders, 132 families, 175 genera, and 163 species were identified. The relative abundance of these phyla from individual samples represented >95% of all sequences. On average, Proteobacteria was the most abundant phylum in all samples, followed by Campylobacterota, Bacteroidota, and other less represented groups, such as Fibrobacterota, Desulfobacterota_I, Firmicutes_D (Figure 3A). Thermal stress induced notable changes in microbial composition. Proteobacteria exhibited a marked decrease in relative abundance under heat stress, declining from 69.83% to 41.56% at week 1 and from 78.12% to 54.68% at week 2. In contrast, Fusobacteriota showed an opposite trend, with a substantial increase from 2.42% to 23.69% at week 1 and from 0.63% to 15.78% at week 2. Conversely, Campylobacterota did not show major variations between treatments, with relative abundances remaining relatively stable (11.86% to 15.59% at week 1 and 13.22% to 11.56% at week 2). (Figure 3A). At the family level, thermal stress induced marked shifts in bacterial composition. Sulfurimonadaceae showed a clear decrease in relative abundance, declining from 37.49% to 15.18% at week 1 and from 48.49% to 32.46% at week 2 (Figure 3B). Similarly, Flavobacteriaceae exhibited a pronounced reduction under heat stress, decreasing from 15.71% to 2.57% at week 1 and from 7.09% to 1.20% at week 2 (Figure 3B). In contrast, Fusobacteriotaceae displayed an opposite trend, with a substantial increase in relative abundance, rising from 12.30% to 58.26% at week 1 and from 4.16% to 43.88% at week 2 (Figure 3B). At the genus level, greater taxonomic resolution was observed, with the identification of specific genera such as *Sulfurimonas*, *Propionigenium,* and other less represented groups, such as *Vibrio*, *Psychromonas*, and *Ferrimonas* (Figure 3C). Differences in the relative abundance of certain genera between treatments are highlighted, which could indicate a specific microbial response to the experimental environment. Specifically, the genus *Sulfurimonas* showed a pronounced decrease under heat stress, declining from 52.62% to 10.16% at week 1 and from 55.19% to 37.37% at week 2 (Figure 3C). Similarly, *Balneicella* exhibited a substantial reduction, from 12.50% to 1.90% at week 1 and from 9.15% to 1.83% at week 2 (Figure 3C). In contrast, *Propionigenium* displayed the opposite trend, with a strong increase in relative abundance under thermal stress, rising from 18.10% to 76.91% at week 1 and from 5.21% to 53.80% at week 2 (Figure 3C).

**Figure 4:**
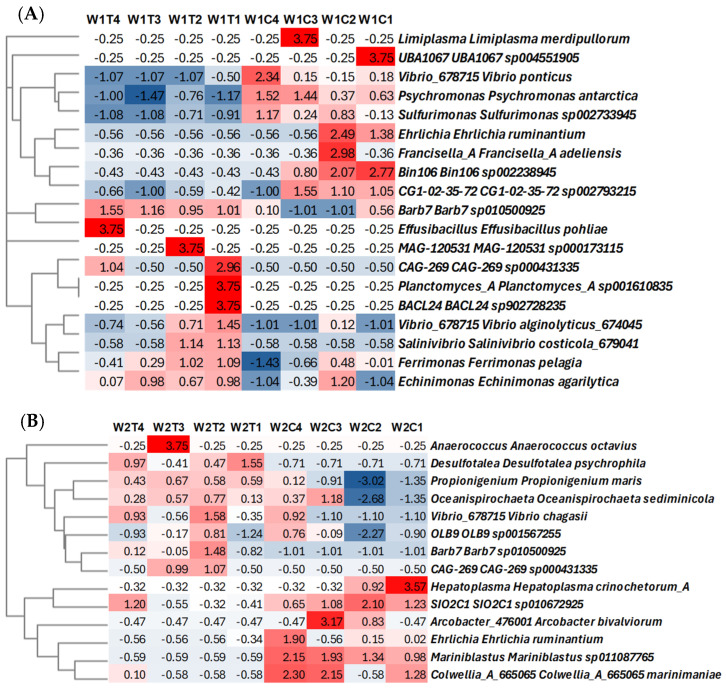
Heat maps with hierarchical clustering analysis to visualize differences in the relative abundance of different bacterial taxa between control and heat stress conditions. (**A**) W1C (control temperature 16 ± 2 °C, week 1), W1T (high-temperature 22 ± 2 °C, week 1). (**B**) W2C (control temperature 16 ± 2 °C, week 2) and W2T (high-temperature 22 ± 2 °C, week 2).

Hierarchical clustering analysis based on heat maps revealed clear differences in the relative abundance of bacterial species between control and heat-stress treatments (Figure 4). During week 1, a clear compositional shift in the bacterial community was observed between the two groups. The control group, dominated by *Psychromonas antarctica*, *Sulfurimonas* sp., *Ehrlichia ruminantium*, and *Francisella_A adeliensis*, was characterized by taxa typically associated with cold environments, sulfur metabolism, and host-associated or intracellular lifestyles. In contrast, the heat stress group, including *Vibrio alginolyticus*, *Salinivibrio costicola*, *Ferrimonas pelagia*, and *Echinimonas agarilytica*, showed a predominance of halophilic and marine heterotrophic bacteria commonly linked to more dynamic and nutrient-rich conditions. Notably, the increased representation of *Vibrio* and *Salinivibrio* suggests a shift toward opportunistic and metabolically versatile taxa, whereas the relative abundance of *Psychromonas* and *Sulfurimonas* in the first group indicates a community more adapted to stable or specialized ecological niches. During week 2, thermal stress induced a clear shift in microbial composition compared to the control. The temperature-treated group was dominated by *Desulfotalea psychrophila*, *Propionigenium maris*, *Oceanispirochaeta sediminicola*, and *Vibrio chagasii*, suggesting increased anaerobic metabolism and opportunistic bacterial proliferation. In contrast, the control condition showed higher abundance of *Arcobacter bivalviorum*, *Ehrlichia ruminantium*, *Mariniblastus* sp., and *Colwellia marinimaniae*, indicative of a more stable and cold-adapted community. These patterns are consistent with the shifts observed in community composition and diversity indices. In summary, thermal stress promoted a marked restructuring of the gut microbiota, characterized by a reduction in dominant taxa associated with microbial homeostasis and an increase in opportunistic bacterial groups. 

### 3.4. Oxidative Damage in Red Sea Urchin Gut

**Figure 5:**
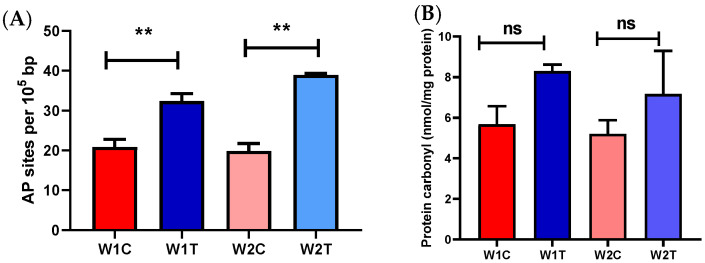
Oxidative damage in *L. albus* gut. (**A**) Levels of apurinic/apyrimidinic (AP) sites per 100,000 bp. (**B**) Protein carbonyl content (nmol/mg protein) among groups W1C (control temperature 16 ± 2 °C, week 1), W2C (control temperature 16 ± 2 °C, week 2), W1T (high-temperature 22 ± 2 °C, week 1), and W2T (high-temperature 22 ± 2 °C, week 2). Results are expressed as means ± standard error (*n* = 4). Asterisks indicate significant differences between the groups (** = *p* < 0.01); ns: non-significant.

Heat stress induced significant oxidative damage to DNA in the gut of *L. albus*. At both sampling points, sea urchins exposed to elevated temperature showed significantly higher levels of AP sites compared to controls (Figure 5A). In contrast, protein carbonyl content in the gut of *L. albus* did not show significant differences between control and heat-stress groups at any time point, suggesting that oxidative DNA damage was more evident than protein oxidation under the experimental conditions evaluated (Figure 5B). Overall, thermal stress induced significant oxidative DNA damage in the gut of *L. albus*, whereas protein oxidation remained largely unaffected.

### 3.5. Immune and Stress-Related Gene Expression Red Sea Urchin Gut

**Figure 6:**
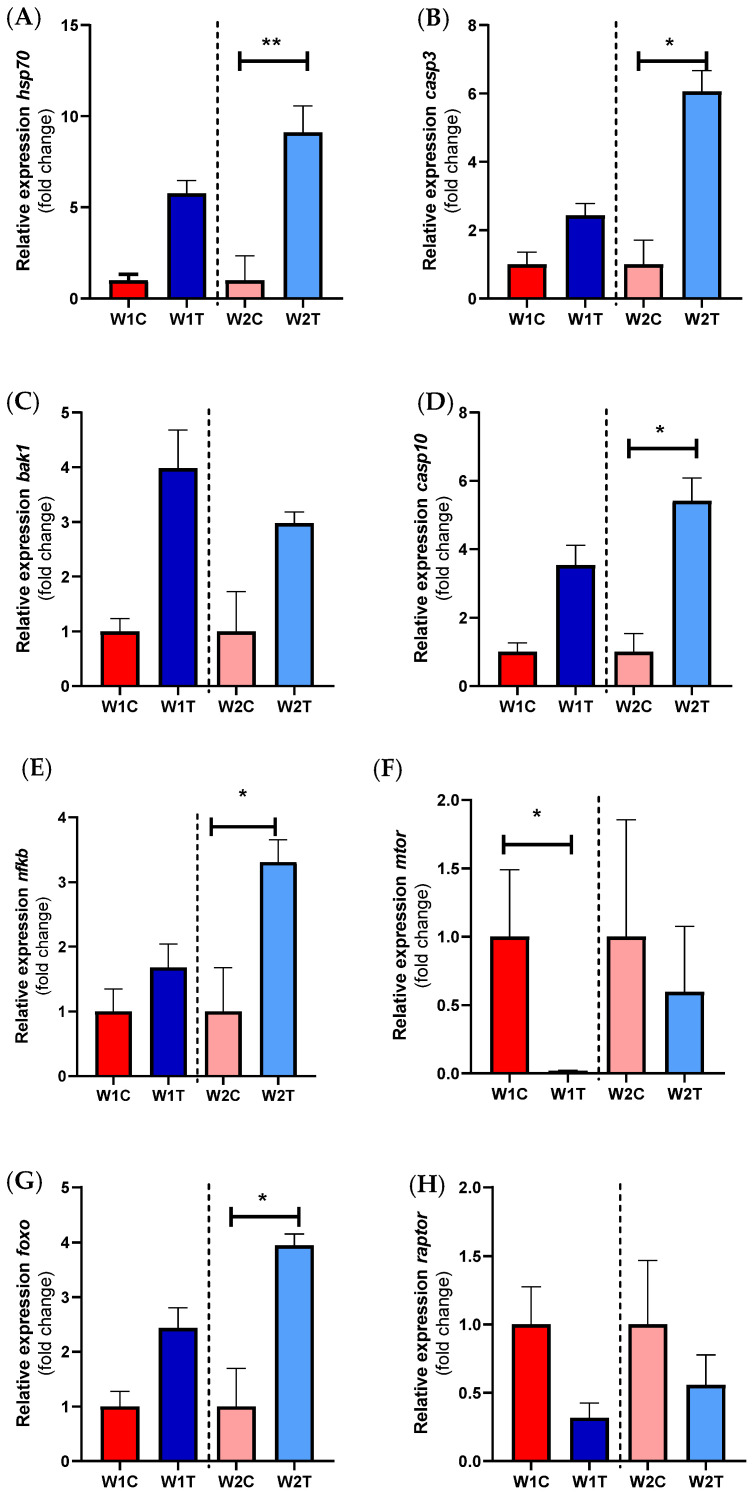
Effect of increased temperature on the expression of genes associated with the heat shock response, apoptosis pathway and cellular signaling in the gut of *L. albus*. The graph shows relative expression in fold change levels of the genes: (**A**) heat shock protein 70 (*hsp70)*, (**B**) Caspase-3 (*casp3*); (**C**) BCL2 Antagonist/Killer 1 (*bak1*), (**D**) Caspase-10 (*casp10*), (**E**) Nuclear factor kappa-light-chain-enhancer of activated B cells (*nfkb*), (**F**) mechanistic Target of Rapamycin (*mtor*), (**G**) Forkhead box O (*foxo*), and (**H**) Regulatory Associated Protein Of MTOR Complex 1 (*raptor*), among groups W1C (control temperature 16 ± 2 °C, week 1), W2C (control temperature 16 ± 2 °C, week 2), W1T (high-temperature 22 ± 2 °C, week 1), and W2T (high-temperature 22 ± 2 °C, week 2). Expression levels were normalized to the 18s rRNA reference gene. Results are expressed as means ± standard error (*n* = 4). Asterisks indicate significant differences between the groups (* = *p* < 0.05; ** = *p* < 0.01).

Thermal stress induced significant changes in the expression of genes associated with cellular stress, apoptosis, and immune regulation in the intestinal tissue of *L. albus*. The expression of *hsp70* was strongly affected by temperature. After two weeks of exposure, heat-stressed individuals showed a significant 9.1-fold increase in *hsp70* expression compared to controls, indicating a cellular stress response (Figure 6A). Genes involved in the apoptotic pathway also exhibited significant modulation in the intestine of *L. albus*, in response to heat stress. At week 2, both *casp3* and *casp10* were significantly upregulated in heat-stressed individuals compared to controls, exhibiting 6.1-fold and 5.4-fold increases, respectively, indicating activation of caspase-dependent apoptosis (Figure 6B,D). In contrast, *bak1* did not show significant differences between treatments at either time point (Figure 6C), suggesting a limited involvement of mitochondrial-mediated apoptotic regulation under the experimental conditions. Similarly, genes associated with the mTOR signaling pathway and immune regulation showed differential responses to thermal stress in the gut of *L. albus*. The transcription factor *nfκb* was significantly upregulated in heat-stressed individuals after two weeks of exposure, showing a 3.3-fold increase relative to controls (Figure 6E), as was *foxo*, which exhibited a 3.9-fold increase (Figure 6G), indicating activation of the immune and oxidative stress response pathway. In contrast, *mtor* showed a significant decrease in expression at week 1, reaching only 0.02-fold of control levels (Figure 6F), while *raptor* did not present significant differences between treatments (Figure 6H). Taken together, these results indicate that thermal stress activated molecular pathways associated with cellular stress, apoptosis, immune regulation, and oxidative stress responses in the gut of *L. albus*.

## 4. Discussion

The present study demonstrates that elevated seawater temperature exerts a profound impact on the feeding, gut microbiome, and molecular stress responses of the red sea urchin under aquaculture conditions. Under control conditions, individuals exhibited higher feeding activity, along with a more diverse gut microbial community dominated by Proteobacteria and Campylobacterota, taxa commonly associated with nutrient cycling and gut homeostasis in marine invertebrates [45,46]. In contrast, heat-stressed individuals showed reduced food intake, marked by a progressive loss of microbial diversity. This shift included an increased relative abundance of opportunistic taxa such as *Vibrio* and *Propionigenium*. These alterations were accompanied by significant oxidative DNA damage in intestinal tissue, indicating increased cellular stress under elevated temperature, whereas protein oxidation remained unchanged. Furthermore, thermal stress induced early upregulation of genes associated with apoptosis (*casp3*, *casp10*), immune regulation (*nfkb*, *foxo*), and cellular stress response (*hsp70*), suggesting activation of molecular defense mechanisms. Collectively, these findings reveal a coordinated, multi-level response to thermal stress involving microbial imbalance, physiological impairment, oxidative damage, and immune activation.

### 4.1. Thermal Stress and Gut Microbiota Imbalance

Rising seawater temperature is primarily associated with global climate change and the increasing frequency, duration, and intensity of marine heatwaves [47]. In coastal benthic organisms such as sea urchins, thermal anomalies can affect distribution, habitat use, feeding activity, reproductive performance, larval development, and interactions with benthic microbial communities [48]. These effects may be particularly relevant for *L. albus*, whose fishery and aquaculture potential depend strongly on the maintenance of suitable environmental conditions along the Chilean coast [49]. Elevated seawater temperature significantly altered both the composition and structure of the intestinal microbiota in *L. albus*, as well as feeding behavior and activity patterns. Under normal culture conditions, sea urchins displayed higher algal consumption and typical behavioral responses, accompanied by a microbiota characterized by high diversity and dominated by taxa beneficial for digestion and immune function, such as Proteobacteria and Campylobacterota [45,46,50]. However, under heat stress, a significant reduction in feeding and reduced microbial diversity consistent with dysbiosis were observed. This reduced microbial diversity was characterized by a decline in key taxa such as Proteobacteria and a concomitant increase in opportunistic or potentially pathogenic bacteria, including *Vibrio* and *Propionigenium*, which have been previously associated with stress and disease in marine invertebrates [51]. These changes suggest a disruption of microbial homeostasis. Notably, this effect became more pronounced during the second week of exposure, indicating that prolonged thermal stress exacerbates microbial imbalance. The observed reduction in feeding activity likely reflects an adaptive reallocation of energy toward survival rather than growth and consumption, a response commonly reported in invertebrates exposed to thermal stress [52,53]. Elevated temperatures are also known to increase heterogeneity in microbial community composition [54]. Consistent with this, both the Shannon index and Simpson index revealed a marked decrease in gut microbial diversity in heat-stressed individuals. Reduced microbial diversity under thermal stress has been widely reported across marine invertebrates, including sea urchins [15,38], sea cucumbers [14], sea stars [55], mussels [18], and oysters [56]. High microbial diversity is generally associated with increased resilience and stability in host organisms, whereas reduced diversity is often linked to compromised health [57]. The present study revealed that the gut microbiota of *L. albus* under normal culture conditions was dominated by two major bacterial phyla, which likely play key roles in gut function. Proteobacteria are commonly found in high abundance in the gut of marine invertebrates, including planorbid snails [58], sea slugs [59], and echinoderms such as the sea cucumber *Apostichopus japonicus* [14] and *L. variegatus* [46]. Campylobacterota, in contrast, are widely distributed across both terrestrial and marine environments and are involved in diverse metabolic functions [50]. In marine systems, they have been reported at high abundance in the gut of *L. variegatus*, reaching up to 92% [15]. Our results indicate that elevated water temperature reduced the relative abundance of Proteobacteria in heat-stressed sea urchins, accompanied by an increase in Fusobacteriota, a shift that may reflect dysbiosis within the gut ecosystem of *L. albus*. Additionally, an increased abundance of *Propionigenium* was observed, a genus recognized for its opportunistic behavior and proliferation under disturbed environmental conditions [60]. Similarly, the presence of bacterial species belonging to the genus *Vibrio* was notable, as these taxa are often dominant in marine environments and have been associated with both healthy and stressed host conditions [61]. Although *Propionigenium* has been reported to increase under disturbed environmental conditions, it is also an anaerobic propionate-producing bacterium that may contribute to host metabolism. Therefore, its increased abundance should not necessarily be interpreted as pathogenic, and may instead reflect shifts in intestinal environmental conditions induced by thermal stress [60,62]. Further investigation is warranted to clarify their role within the sea urchin gut microbiome. Specifically, it remains to be determined whether their increased abundance serves as an indicator of stress or disease, or whether they represent stable members of a microbial reservoir that become more prominent under host-compromised conditions. As observed in other echinoderms and marine invertebrates [55], these taxa may constitute an active component of the holobiont that rapidly expands in response to environmental or physiological disturbance. Although several bacterial taxa were assigned at the species level, these classifications should be interpreted with caution because they were inferred from short-read sequencing of the V4 region of the 16S rRNA gene, which provides limited taxonomic resolution for distinguishing closely related species.

### 4.2. Oxidative Damage and Cellular Stress Responses

Heat-stressed individuals exhibited a significant increase in oxidative DNA damage, as indicated by elevated levels of AP sites at both sampling points. This finding reflects enhanced production of ROS and suggests that prolonged exposure to elevated temperatures compromises genomic integrity in intestinal tissues. The progressive increase in DNA damage over time further supports the presence of a cumulative oxidative burden under sustained thermal stress. Similar patterns of oxidative DNA damage under thermal or environmental stress have been reported in other marine organisms, including bivalves such as *Mytilus galloprovincialis*, where elevated temperatures promote ROS production and genomic instability [63]. In fish, thermal stress has also been linked to oxidative damage in nucleic acids, which are considered primary targets of ROS [21]. In contrast, protein carbonylation levels in intestinal tissue did not differ significantly between control and heat-stressed groups. This suggests that proteins may be less susceptible than DNA to oxidative damage under experimental conditions, or that antioxidant defense mechanisms are sufficiently effective to preserve protein integrity despite increased ROS production. This differential sensitivity among biomolecules has been observed in other marine species, where nucleic acids often represent earlier and more sensitive targets of oxidative stress compared to proteins [22]. In addition to oxidative damage, thermal stress induced significant modulation of genes associated with apoptosis, immune response, and cellular stress pathways. The upregulation of *casp3* and *casp10* during early exposure indicates activation of caspase-dependent apoptotic processes, which are essential for the removal of damaged cells and the maintenance of tissue integrity under stress conditions [23]. Supporting this, studies in sea urchins have demonstrated that thermal stress can increase apoptotic activity in gonadal tissues, as observed in juveniles of the Atlantic sea urchin *Arbacia punctulata* exposed to elevated temperatures over short-term periods [64]. Because only selected apoptotic markers were evaluated, the involvement of alternative apoptotic pathways, including receptor-mediated mechanisms, cannot be excluded. The absence of significant changes in *bak1* suggests that apoptosis in this context may be primarily regulated through executioner caspases rather than mitochondrial-mediated pathways. In contrast, the strong induction of *hsp70* further supports the presence of acute cellular stress, as heat shock proteins function as molecular chaperones that stabilize protein structure and prevent aggregation under adverse environmental conditions [65]. In sea urchins, *hsp70* is widely recognized as a sensitive biomarker of environmental stress, particularly in immune cells such as coelomocytes, where its expression rapidly increases in response to thermal and chemical stressors [29]. This rapid transcriptional activation reflects an early protective mechanism aimed at maintaining protein homeostasis under stress conditions. The observed upregulation in *L. albus* is consistent with this role and suggests an immediate cellular response to elevated temperature. Moreover, this response aligns with the increased oxidative DNA damage observed, indicating that intestinal cells activate protective mechanisms to counteract ROS-induced damage during the initial phase of thermal exposure.

Moreover, the upregulation of *nfκb* and *foxo* suggests activation of immune and oxidative stress response pathways. NFκB is a central regulator of innate immunity and inflammatory responses, whereas FOXO transcription factors are involved in oxidative stress resistance, apoptosis regulation, and metabolic homeostasis [26,66,67]. In marine invertebrates, activation of NFκB signaling has been associated with environmental stress and immune modulation, as observed in bivalves exposed to acidification, where this pathway is significantly upregulated under stress conditions [26]. In contrast, FOXO plays a key role in regulating antioxidant defenses by controlling the expression of enzymes such as superoxide dismutase and catalase, which are essential for ROS detoxification. The upregulation of *foxo* observed in this study likely represents an early compensatory response aimed at mitigating oxidative damage. However, as suggested by previous studies, this response may be insufficient under sustained stress conditions, leading to the accumulation of oxidative damage when antioxidant mechanisms are overwhelmed [68]. This interpretation is consistent with the increased DNA damage observed here, indicating that although antioxidant pathways are activated, they may not fully counteract the effects of prolonged thermal stress. Conversely, the limited response of *mtor* and *raptor* suggests that metabolic signaling pathways may be less sensitive to short-term thermal stress or may operate over different temporal scales. The mTOR signaling pathway is known to regulate cell growth, protein synthesis, and energy balance in response to environmental and nutritional cues [69]. In the green sea urchin *Strongylocentrotus droebachiensis*, this pathway has been primarily associated with developmental processes such as larval growth and metamorphosis, rather than acute stress responses [70]. Interestingly, although *mtor* expression showed limited modulation, the increased oxidative DNA damage suggests that thermal stress may affect cellular energy-sensing pathways [71]. Elevated ROS levels can suppress mTOR signaling, promoting adaptive responses such as apoptosis and autophagy under stress conditions. Although autophagy markers were not evaluated in this study, the concurrent increase in oxidative damage and apoptotic gene expression is consistent with a shift in cellular resources from growth toward maintenance and survival [72]. The coexistence of gut microbiota reduced microbial diversity, oxidative stress, and immune activation indicates a tightly interconnected response to thermal stress. Microbial imbalance may promote increased ROS production and immune stimulation, while oxidative stress can further disrupt microbial community structure, creating a feedback loop that amplifies physiological stress. Although the present study demonstrates concurrent reduced microbiota diversity and oxidative DNA damage, the experimental design does not allow direct inference of causality between these processes. Nevertheless, previous studies suggest that thermal stress can simultaneously alter microbial community composition and host redox homeostasis. Future studies combining microbiome manipulation approaches and metabolomic analyses will be required to determine whether microbiota changes directly contribute to oxidative stress responses in *L. albus*.

## 5. Conclusions

This study demonstrates that thermal stress significantly disrupts the physiological and microbial homeostasis of *L. albus* under aquaculture conditions. Elevated temperature reduced feeding activity and altered gut microbiota composition, leading to decreased microbial diversity and a shift toward opportunistic taxa. At the cellular level, heat stress induced a marked increase in oxidative DNA damage, reflecting enhanced reactive oxygen species (ROS) production and compromised genomic integrity, while protein oxidation remained unchanged. In parallel, the upregulation of genes associated with apoptosis (*casp3*, *casp10*), immune response (*nfκb*, *foxo*), and cellular stress (*hsp70*) indicates activation of early defense mechanisms aimed at preserving cellular function. The combined occurrence of microbial imbalance, oxidative stress, and immune activation reveals a coordinated, multi-level response to elevated temperature. These interconnected processes likely contribute to the observed physiological impairments and reduced performance. Overall, these findings provide important insights into the mechanisms underlying thermal stress responses in *L. albus*, highlighting its sensitivity to environmental changes. From an applied perspective, this study underscores the need for temperature management strategies in aquaculture systems to enhance resilience and ensure the sustainability of sea urchin production under climate change scenarios.

## Data Availability

The raw read sequences obtained from sequencing were deposited in the Sequence Read Archive (SRA) under BioProject accession number PRJNA1462746. The datasets generated and analyzed during the current study are publicly available.

## Supplementary Materials

The following supporting information can be downloaded at: https://www.mdpi.com/article/10.3390/biology15120913/s1, Table S1: Summary of experimental groups, sampling times, tank replication, and water quality parameters used during the thermal stress experiment; Table S2: List of primers used in real-time PCR for gene expression analysis; Table S3: Summary of 16s RNA sequencing for the red sea urchin microbiome. bp: base pair.
